# Effects of Calcium Salts on the Physicochemical Quality of Cured Beef Sausages during Manufacturing and Storage: A Potential Calcium Application for Sausages with Alginate Casings

**DOI:** 10.3390/foods10112783

**Published:** 2021-11-12

**Authors:** Xiaoyin Yang, Joseph G. Sebranek, Xin Luo, Wangang Zhang, Mengmeng Zhang, Baochen Xu, Yimin Zhang, Rongrong Liang

**Affiliations:** 1 Laboratory of Beef Processing and Quality Control, College of Food Science and Engineering, Shandong Agricultural University, Tai’an 271018, China; yangxiaoyin@sdau.edu.cn (X.Y.); luoxin@sdau.edu.cn (X.L.); xc@sdau.edu.cn (M.Z.); xvbaoo0@gmail.com (B.X.); ymzhang@sdau.edu.cn (Y.Z.); 2 National R & D Center for Beef Processing Technology, Tai’an 271018, China; 3 Department of Animal Science, Iowa State University, Ames, IA 50011-3150, USA; sebranek@iastate.edu; 4 Department of Food Science and Human Nutrition, Iowa State University, Ames, IA 50011-3150, USA; 5 Key Laboratory of Meat Products Processing, Ministry of Agriculture, Jiangsu Collaborative Innovation Center of Meat Production and Processing, Quality and Safety Control, College of Food Science and Technology, Nanjing Agricultural University, Nanjing 210095, China; wangang.zhang@njau.edu.cn

**Keywords:** beef sausages, manufacturing and storage, calcium chloride, calcium lactate, meat color

## Abstract

The impacts of adding calcium chloride (CaCl_2_) and calcium lactate (CaLac) with different concentrations (0%, 0.2%, 0.4%, and 0.7%) on the physicochemical properties of cured beef sausages were investigated in this study. Meat color, pH, lipid oxidation, and cooking loss were measured at respective manufacturing stages (ground beef, raw chopped batter, and after cooking). Additionally, meat color, pH, lipid oxidation, nitrosylhemochrome, residual nitrite, and texture profiles of vacuum-packaged sausages were evaluated during seven days of storage. Compared with the control (no Ca added), both calcium salts resulted in deteriorative color and texture properties, and promoted pH decline, cooking loss, and lipid oxidation of sausages during manufacturing and storage. However, increased calcium salt addition led to the reduction of residual nitrite over time. Compared to CaCl_2_ addition, 0.2–0.4% CaLac resulted in greater redness and oxidative stability and softer texture. These results may be useful when considering calcium salt additions in sausages, for the purpose of co-extruded sausages coated with alginate where Ca salts are used to form the casing during the co-extrusion of the sausages.

## 1. Introduction

Meat manufacturers are continuously developing new technologies to improve the quality and productivity of their products. Sausages are usually made by stuffing the meat batter into natural or artificial casings. As an emerging substitute for traditional casings, co-extruded casings formed from collagen or alginate are becoming popular [1,2]. This technology can be used in the production of various sausage types, such as frankfurters, grilling sausages, and dry fermented sausages, and it has been employed for one third of small-diameter sausages in the U.S. [1,2]. Alginate casings are formed by the cross-linking of alginates by exposure to calcium cations to create thin, elastic, and strong thermo-stable gels, which can be applied on the outside surface of the sausage to form a product casing [3]. Co-extrusion technology is now widely used for continuous sausage production lines with extremely low yield loss and high labor cost savings [2]. However, in the actual manufacturing of alginate-based co-extruded sausages, there have been concerns about the reduced intensity of surface color compared to other casing systems for cured meat products. Given that the final color of cooked meat products primarily depends on the state of myoglobin and myoglobin thermal stability [4], factors that influence the biochemical changes in myoglobin may account for this phenomenon.

Calcium plays a fundamental role in processing co-extruded casings. The co-extruded sausage passes through a CaCl_2_ bath to form a stable polymer film in practice. Thus far, co-extrusion research on alginate gels has been focused on the white efflorescence sometimes induced by calcium immersion solutions [5]. To stabilize the alginate film, a low concentration (about <1%) of calcium salts, such as CaCl_2_, CaLac, calcium acetate, etc., is generally introduced into the batter systems to fortify the gel [1,6]. It has been reported that the addition of calcium salts to sausages can affect their quality. Previous studies have reported on the use of calcium salts as nutrient fortifiers [7,8] or as NaCl substitutes for low-sodium meat products [9]. However, very little information is available on the effects of different low levels of calcium salts on the physicochemical quality of meat products.

Previous research on calcium additions has been conducted primarily in two fields, (1) fresh uncured meat with calcium salts added to improve quality traits and (2) cooked or ripened meat products with calcium salts added to improve nutritional values. For fresh meat, it has been reported that injections of CaCl_2_ accelerated the tenderization of beef, however, at a cost of adverse effects on some other quality traits, such as drip loss, color stability, oxidative stability, and flavors [10,11,12]. Thus, some researchers have studied CaLac instead of CaCl_2_ to overcome those defects, and they found that enhancing beef with CaLac solutions elevated meat color stability and improved beef flavor compared to CaCl_2_ treatments [12,13]. On the contrary, Seyfert et al. [14] found that CaLac decreased the color stability of ground beef and attributed this to an oxidative catalyzing characteristic of CaLac, especially in minced meats. However, the impact of calcium salt additions on the quality of cured meat products is unclear and deserves further study. Given that Ca salts are used to form co-extruded alginate casings during processing, it seems important to investigate meat quality changes with different calcium salts at a range of concentrations in cured meats.

For cooked or ripened meat products, the cooking yield and pH of cooked restructured buffalo meatloaf also decreased significantly with an increased concentration of CaLac [15]. Moreover, the pH, cooking loss, lightness, and overall acceptability of fat-reduced emulsion-type pork sausages gradually decreased when the amount of CaCl_2_ substitution of NaCl increased from 5% to 25% [9]. However, few changes were observed in the texture, meat color, or sensory properties of cooked fermented sausages enriched with CaLac, calcium gluconate, or calcium citrate [7]. In general terms, the addition of calcium salts has shown various influences on the quality of meat products and seems to depend on the type and amount of calcium salts, as well as the type of meat product.

The overall purpose of this work was to determine the effects of varying amounts of CaCl_2_ and CaLac on the physicochemical characteristics of beef sausages during processing and storage. The results are expected to provide for the improvement of Ca applications to meat products, particularly for the consideration of co-extrusion of alginate casings for sausage production.

## 2. Materials and Methods

### 2.1. Experimental Design and Sausage Manufacture

**Figure 1:**
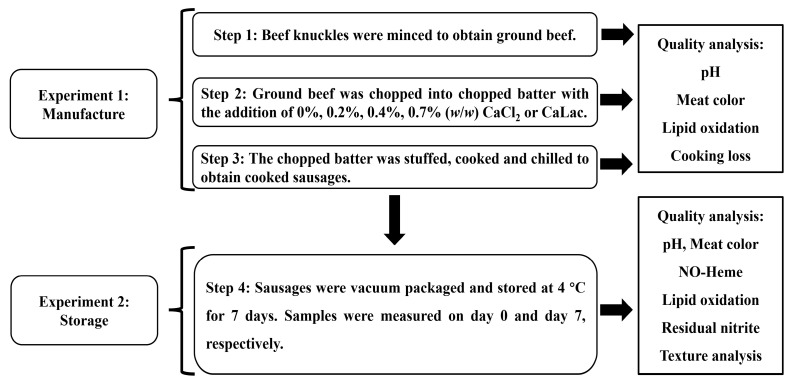
Flow chart of the experimental design and analyzed indicators.

The experiment was independently carried out in triplicate. Each experiment consisted of two separate sections, sausage manufacture and sausage storage, and the experimental design is shown in Figure 1. For the sausage manufacturing process (Experiment 1), frozen beef knuckles were collected in a commercial abattoir from two crossbred Luxi cattle (20–22 months old, 270–300 kg carcass weights) and were allowed to thaw at 4 °C for 48 h. The visible connective tissue was trimmed off, and the thawed meat was ground through a 3-mm steel plate to obtain ground beef using a meat mincer (BJRJ-22T, Aibo Technology Engineering Co., Ltd., Hangzhou, China). The ground beef was separated into seven portions, including three CaCl_2_ groups (0.2%, 0.4%, and 0.7%; *w*/*w)*, three CaLac groups (0.2%, 0.4%, and 0.7%, *w*/*w*), and one control group (no Ca added; 0%, *w*/*w*). Both calcium salts were >98% pure, based on the anhydrous substances (Ruipu Biotechnology Co., Ltd., Zhengzhou, China). Brines were prepared with different Ca additions aforementioned, 1.9% NaCl (*w*/*w*), 0.04% Na-ascorbate (*w*/*w*), and 0.015% NaNO_2_ (*w*/*w*) of the total weight of the formulation. Ground beef, brines, and a water/ice mixture (a final water content at 14% of the total weight of the formulation; *w*/*w*) was chopped in a bowl chopper (K15E, TALSA Co., Valencia, Spain) until a temperature of 12 °C was achieved to obtain the chopped batter. The resulting batter was immediately loaded into a vacuum stuffer (F-line F50, Frey, Herbrechtingen, Germany), stuffed into 28-mm diameter artificial plastic casings, and linked at about 15-cm intervals. Each sausage was weighed (about 90 g) and then placed in 85 °C water to reach a central temperature of 72 °C of the sausage. After cooking, the products were cooled in an ice-water bath to a core temperature of 4 to 6 °C. Meat color, pH, and lipid oxidation were evaluated on the ground beef, raw chopped batter, and cooked sausages after chilling, respectively. The cooking loss of cooked sausages was also measured after the removal of the casing.

For the sausage storage process (Experiment 2), the sausage produced in session 1 was vacuum packaged into polyethylene bags using a Multivac C200 (Multivac Sepp Haggenmüller GmbH & Co. KG, Wolfertschwenden, Germany) and stored at 4 °C for seven days. At the end of storage, six sausages were randomly selected from each group for a physicochemical analysis (meat color, pH, lipid oxidation, residual nitrite, and NO-heme contents) and texture analysis, respectively. Likewise, six sausages prior to packaging were randomly selected from each group to analyze the aforementioned indicators as initial data on day 0.

### 2.2. Methods

#### 2.2.1. pH

Samples (10 g) were homogenized in a blender with 90 mL of distilled water. The mixture was filtered through a filter paper (150, Hangzhou Special Paper Industry Co., Ltd., Fuyang China) and was then measured with a pH meter (SevenMulti, Mettler-Toledo, Schwerzenbach, Switzerland). The average of the triplicate measurements was obtained.

#### 2.2.2. Cooking Loss

The total weight of raw sausage was recorded as W_1_. After the sausage was cooked and stored at 4 °C for 24 h, its casing was stripped, and the surface and casing were patted dry with paper towels. The cooked sausage and casing were reweighed as W_2_ and W_0_, respectively. Cooking loss (%) = (W_1_ − W_2_ − W_0_)/(W_1_ − W_0_) × 100.

#### 2.2.3. Instrumental Color

The lightness (L*), redness (a*), and yellowness (b*) values of ground beef, raw chopped batter, and a cross section in the center of the sausage links were scanned using an X-Rite SP62 spectrophotometer (4 mm diameter aperture, Illuminant A, 10° observer, Grand Rapids, MI, USA). The average of triplicate color measurements of each sample was recorded. Chroma was also calculated using the equation (a*^2^ + b*^2^)^1/2^. Hue was obtained from arc tan (b*/a*).

#### 2.2.4. Thiobarbituric Reactive Substances (TBARS)

Lipid oxidation was analyzed according to a modified method of Draper et al. [16]. The sample (4 g) was homogenized with 20 mL of 10% trichloroacetic acid plus 2 mL of 500 ppm butylated hydroxytoluene and 0.4 mL of 0.5% sulfonamide using an Ultra Turrax homogenizer (T18, IKA, Staufen, Germany) for 2 min. The homogenate was filtered with Whatman NO.1 filter paper. The 0.3 mL of filtrate was added into 0.6 mL of 0.02 M thiobarbituric acid (TBA) in a 2 mL centrifuge tube. The tube was incubated in a metal bath at 80 °C (DKT 200-2, Hangzhou, China) for 90 min. After cooling, the absorbance was measured at 532 nm against a TBA blank using a spectrophotometer (BioTek Epoch2, BioTek Instruments, Inc., Winooski, VT, USA). The TBARS values were calculated by a standard prepared from malonaldehyde with 1, 1, 3, 3-tetraethoxypropane (TEP) and expressed as MDA mg/kg meat.

#### 2.2.5. Residual Nitrite

Residual nitrite contents were determined as described by Patton [17]. The extracted nitrite ion from minced samples was reacted with sulfanilamide and the Greiss reagent (sulfanilamide + N-(1-napnhyl)-ethylenediamine, NED) via a spectrophotometric measurement. The absorbance was read at 540 nm, and then nitrite values were calculated from a prerecorded standard curve. Duplicate measures were averaged for each sample.

#### 2.2.6. Measurement of Nitrosylhemochrome (NO-heme)

The pigment NO-heme content of cooked sausages was measured in accordance with the method described by Hornsey [18]. All the procedures were conducted under anaerobic condition in a dark room illuminated by a weak red light at room temperature. All solvents were deoxygenated by flushing with a stream of nitrogen gas. The minced sample (10 g) was mixed with 40 mL of acetone and 3.2 mL of water. The mixture was stirred intermittently for 5 min and then centrifuged (1000× *g*, 5 min). Absorbance of the supernatant was measured at 540 nm against the acetone/water blank. The NO-heme content was calculated by the formula NO-heme (mg/kg) = A_540_ × 290.

#### 2.2.7. Texture Analysis (TPA)

Texture analysis was performed according to the method of Zhao et al. [19], with a slight modification at room temperature. Three cylindrical samples (28 mm diameter × 20 mm height) were cut from the center of the sausage to determine hardness (N; maximum force required to compress the sample), springiness (mm; ability of sample to recover its original shape after a deforming force was removed), cohesiveness (extent to sample that could be deformed prior to rupture), gumminess (N; force necessary to disintegrate a semisolid sample for swallowing), chewiness (N; work to masticate the sample for swallowing), and resilience (force of sample to regain its original shape following the first compression). The TPA of sausages was measured using a TA-XT Texture Analyzer (Stable Micro Systems Ltd., Godalming, UK) with an aluminum cylindrical probe (SMP P/50). The trigger force used for the test was 5 g. Samples were placed on a platform with a fixture and then compressed twice to 50% of their original height with a pretest speed and a test speed at 2.0 mm/s, respectively, and a post-test speed at 5.0 mm/s. The data were generated by Exponent software (Exponent Stable Microsystem, version 5.1.2.0, Stable Microsystems Ltd., Godalming, UK).

### 2.3. Statistical Analysis

The data were analyzed using the MIXED procedure of SAS (V 9.0, SAS Institute Inc., Cary, NC, USA). For meat color, pH, and lipid oxidation during the sausage processing, calcium additions (CaCl_2_ and CaLac), salt concentrations (0%, 0.2%, 0.4%, and 0.7%), process steps (ground beef, raw chopped batter, and cooked sausage), and their interaction were regarded as fixed factors, and experimental batches were regarded as a random factor. For meat color, pH, lipid oxidation, residual nitrite, NO-heme, and texture profile during storage, batches were again considered to be the random effect, with salt and salt concentrations, storage time (0 and 7 days), and their interaction considered as fixed factors. Least squares mean was generated for the fixed effects and their interaction using the PDIFF option, and a significant difference was considered at *p* < 0.05.

## 3. Results and Discussion

### 3.1. Experiment 1: Effects of Calcium Salts on the Quality of Beef Sausages during Processing

#### 3.1.1. pH and Cooking Loss Changes

There was a significant salt type × salt concentration × processing interaction for pH values. Both CaCl_2_ and CaLac additions significantly reduced the pH of the raw chopped batter and cooked sausages (Table 1). This is in agreement with Horita et al. [20], who found that the elevated CaCl_2_ additions in salt blends resulted in a decrease in the pH of both the batter and the final reduced-sodium frankfurter products (*p* < 0.05). Similarly, there was also a significant drop (*p* < 0.01) in the pH of restructured buffalo meat loaves with the increasing CaLac concentration [15].

Compared to ground beef without calcium salts, the raw chopped batter with CaCl_2_ or CaLac additions showed lower (*p* < 0.05) pH values, but the pH values significantly increased for sausages with low levels of CaCl_2_ (0–0.2%) and all levels of CaLac after heating (Table 1). A gradually increased pH value that occurred in the heating process of beef muscle was also reported by Laakkonen et al. [21]. This may be caused by the heat-induced dynamic changes of acidic and basic groups in denatured proteins. It is noteworthy that both the chopped batter and cooked sausages containing CaCl_2_ exhibited lower (*p* < 0.05) pH values than those containing CaLac, which may further lead to the differences in other attributes of sausages with various calcium salt additions. Such differences in pH values may be attributed to the lower pH of CaCl_2_ treated brines than to that of CaLac treated brines from the perspective of the ionization equilibrium of both Ca salt solutions.

**Figure 2:**
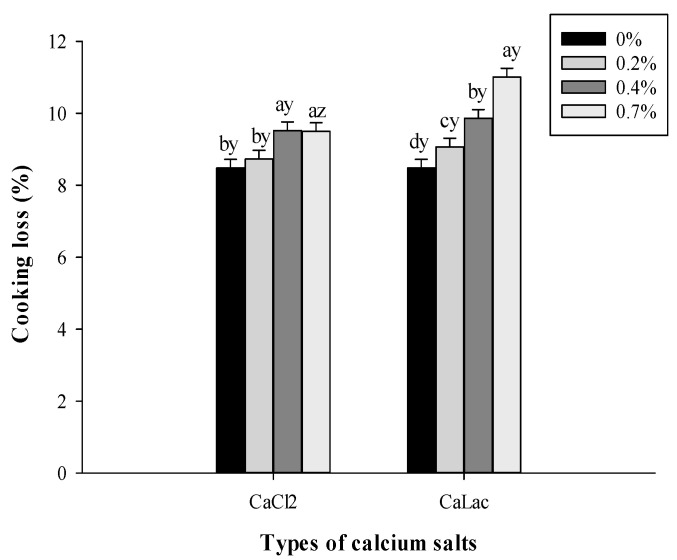
Effects of calcium salt types and concentrations on the cooking loss of beef sausages. ^a–d^ Means within the same calcium salt type with different superscript letters differ (*p* < 0.05). ^y,z^ Means within the same calcium salt concentration with different superscript letters differ (*p* < 0.05).

A significant salt type and salt concentration interaction were observed for cooking loss. As shown in Figure 2, the cooking loss of 0.4–0.7% CaCl_2_ treated sausages were higher (*p* < 0.05) than those of low level CaCl_2_ (0–0.2%) treated sausages. Similarly, an increased CaCl_2_ substitution caused gradual increases in the cooking loss of pork sausages [9]. In addition, sausages displayed a significantly increased cooking loss as the CaLac level increased, and 0.7% CaLac treated sausages manifested a higher (*p* < 0.05) cooking loss than 0.7% CaCl_2_ treated sausages. Considering that the weak hydration of the myofibrillar proteins at their iso-electric point in low pH muscle accounts for greater water losses on cooking [22], the lower pH in both calcium treatments promoted the higher cooking loss for sausages. On the other hand, the lowest water-holding capacity in cooked chicken patties containing CaLac was also reported by Naveena et al. [23], who attributed this to a lower water binding ability caused by the increasing number of tightly bound multivalent cations [22].

#### 3.1.2. Color Analysis

There was a significant salt type × salt concentration × processing interaction for L*, a*, b*, chroma and hue values. Compared with ground beef, the L* values gradually increased (*p* < 0.05) in the chopped batter and subsequent cooked sausages for both CaCl_2_ and CaLac additions at each level (Table 2), however, b* and Chroma values gradually declined (*p* < 0.05). The a* values declined (*p* < 0.05) first in the raw chopped batter and then increased (*p* < 0.05) in the cooked sausage, whereas hue values showed an opposite trend to a* values. The decline in a*, b*, and chroma values and the increased hue values in the raw chopped batter are associated with the addition of NaNO_2_. A rapid browning will appear when nitrite-containing brines are added to fresh meats because muscle pigments are oxidized to the brown metmyoglobin (MMb) by NaNO_2_, and they simultaneously form brown nitrosometmyoglobin [24]_._ The NO-myoglobin is denatured upon cooking and is converted to pink-colored nitrosylhemochrome [25], resulting in a significant increase in the a* values of cooked sausages.

There was a significant decrease in L*, a*, and chroma values of cooked sausages with the increasing CaCl_2_ level ranging from 0.2% to 0.7%, which is concomitant with the increased hue values. Similar color trends that occurred in the chopped batter probably contributed to these color changes in cooked sausages. This is in accordance with Lawrence et al. [12], who reported high levels of CaCl_2_-injection induced muscle darkening and faster discoloration (declining redness) compared with low levels of CaCl_2_-injection. More precisely, CaCl_2_ accelerated an oxidation of haem pigments with the consequence that the meat surface turned brown faster [26]. There were also significantly decreased a* and chroma values and increased hue values in the raw chopped batter with increasing CaLac concentrations, while no differences in a*, chroma, and hue values among cooked sausages with various CaLac concentrations were detected in this study (Table 2). Therefore, CaLac had little effect on the meat color of cooked sausages (*p* > 0.05). Analogous results were reported by Cáceres et al. [7] and Daengprok et al. [8].

Previous studies have demonstrated that CaLac enhancements resulted in raw meat with less MMb and higher a* than other calcium treatments due to the conversion from lactate to NADH and pyruvate by lactate dehydrogenase [12,27]. Contradictory to those reports, no color improvements of CaLac on the raw chopped batter were found in our results, which may be due to the biochemical response time being quite short. Furthermore, the reduced pH in the raw chopped batter caused by increasing levels of both calcium salts would facilitate the heme oxidative browning in the presence of nitrite [28]. It is worth noting that cooked sausages with 0.4–0.7% CaLac had higher a* and chroma values and lower hue values than cooked sausages with the same CaCl_2_, indicating that cooked sausages with 0.4–0.7% CaLac retained red color better than cooked sausages with same levels of CaCl_2_.

#### 3.1.3. Lipid Oxidation

**Figure 3:**
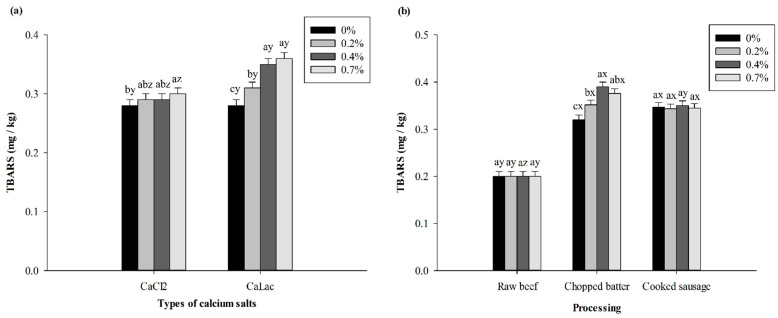
Effects of calcium salt concentrations and calcium salt types (**a**) or processing treatments (**b**) on TBARS values during the processing of beef sausages. ^a–c^ Means within the same calcium salt type or processing treatment with different superscript letters differ (*p* < 0.05). ^x–z^ Means within the same calcium salt concentration with different superscript letters differ (*p* < 0.05).

Both the interactions of salt concentration with salt type and processing impacted the TBARS values (*p* < 0.05). The TBARS values of treatments containing both calcium salts increased significantly as salt concentrations increased (Figure 3a), and treatments with CaLac resulted in higher (*p* < 0.05) TBARS values than treatments with CaCl_2_. Moreover, for both calcium salt treatments, the TBARS values of raw chopped batter and cooked sausages were higher (*p* < 0.05) than those of ground beef (Figure 3b). This is in agreement with Lawrence et al. [12], who pointed out that CaCl_2_ and CaLac injection treatments enhanced the lipid oxidation of beef strip loins during 14 days of postmortem aging.

### 3.2. Experiment 2: Effects of Calcium Salts on the Quality of Beef Sausages during Storage

#### 3.2.1. pH Changes

**Figure 4:**
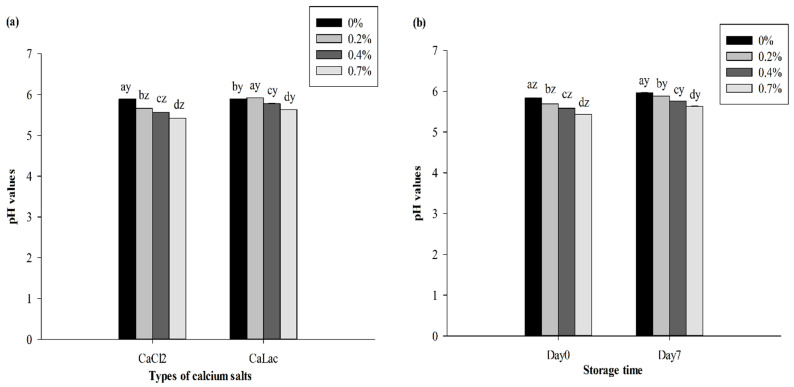
Effects of calcium salt concentrations and calcium salt types (**a**) or storage time (**b**) on the pH values of beef sausages during chilled storage. ^a–d^ Means within the same calcium salt type or storage time with different superscript letters differ (*p* < 0.05). ^y–z^ Means within the same calcium salt concentration with different superscript letters differ (*p* < 0.05).

Both the interaction of salt concentration with salt type and with storage time influenced pH values (*p* < 0.05). The pH of sausages decreased (*p* < 0.05) with increases in both salt concentrations during storage, though CaCl_2_ treated sausages had a faster decline rate in pH than CaLac treated sausages (Figure 4a). Similarly, Kim et al. [9] reported a negative (*p* < 0.01) correlation between pH and CaCl_2_ substitution levels in fat-reduced emulsion-type pork sausages and found that the pH was gradually decreased with increasing CaCl_2_ levels. Regardless of the salt type, similar trends with salt concentrations also occurred on both day 0 and day 7, and the pH of sausages with each salt concentration on day 7 was higher (*p* < 0.05) than the initial pH on day 0 (Figure 4b). The reduced pH in cooked pork rolls containing CaLac was also reported by Devatkal et al. [27], who observed a significant increase in pH over time and attributed this to a degradation of proteins and production of amines.

#### 3.2.2. Color Analysis

There was a significant salt type × salt concentration × storage time interaction for L*, a*, b*, and hue values. The L* values for sausages with the calcium salt additions and without calcium salts all increased significantly during storage (Table 3). It has been reported that the addition of calcium salts (calcium carbonate and calcium citrate malate) lightened the color of frankfurter sausages [29]. However, the calcium salt additions in this study did not change the L* values of sausages during storage, except for the 0.4% additive level. It is noteworthy that 0.4% CaCl_2_ or 0.4% CaLac treated sausages resulted in higher (*p* < 0.05) L* values than sausages with other additive levels on the last day. With the salt level increased from 0.2% to 0.7%, sausages containing CaCl_2_ showed a more pronounced decline in a* and b* values than sausages containing CaLac at the end of storage. By contrast, sausages with CaCl_2_ significantly raised hue values as increasing levels, and their values with 0.7% CaCl_2_ were higher (*p* < 0.05) than those of sausages with 0.7% CaLac on day 7. Kim et al. [9] also found a gradual degradation in the color of pork sausages when CaCl_2_ addition levels were above 5%. Meanwhile, 0.2–0.7% CaLac resulted in higher (*p* < 0.05) a* and b* values compared to the same levels of CaCl_2_. Likewise, Naveena et al. [23] reported higher a* and b* values in cooked chicken patties containing 0.25% CaLac when compared to the control.

Chroma, indicating the color intensity, was significantly influenced by an interaction between salt type and salt concentration. There were gradually decreased chroma values for the sausages as CaCl_2_ levels increased from 0.2–0.7% (*p* < 0.05), and the chroma values were lower (*p* < 0.05) than those for 0.2–0.7% CaLac treated sausages. Taken together, our results indicate that CaLac additions provided a redder sausage color than CaCl_2_ during sausage storage.

**Figure 5:**
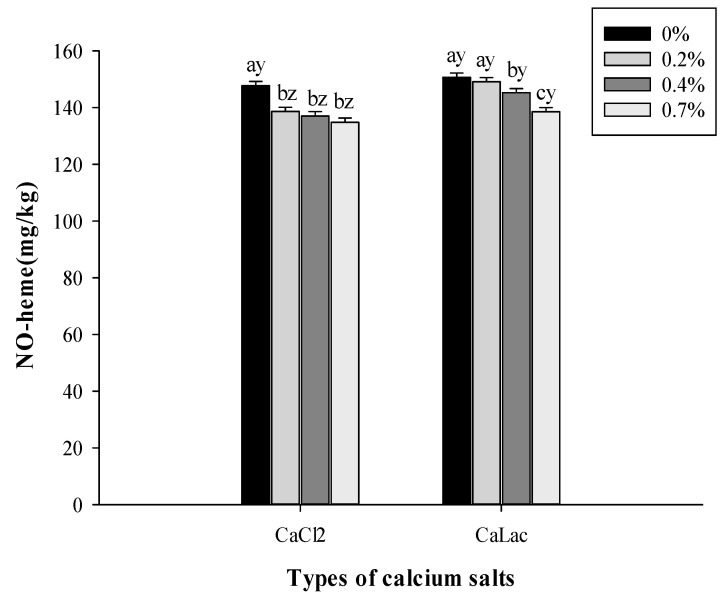
Effects of calcium salt concentrations and calcium salt types on the NO-heme content of beef sausages during chilled storage. ^a–c^ Means within the same calcium salt type with different superscript letters differ (*p* < 0.05). ^y,z^ Means within the same calcium salt concentration with different superscript letters differ (*p* < 0.05).

The NO-heme is a primary red pigment in cooked cured meat, and its contents determine the degree of redness. Salt type and salt concentration interacted (*p* < 0.05) to affect NO-heme contents. Compared to the control, adding 0.2–0.7% CaCl_2_ significantly reduced the NO-heme contents of sausages (Figure 5). A pronounced decline (*p* < 0.05) in NO-heme contents was also noted as an increased level of CaLac from 0.2% to 0.7%. These declines in NO-heme contents for both Ca salts treated sausages may be caused by the gradually decreased pH values with higher levels of Ca salt additions. Yu et al. [30] explained that weakly acidic conditions were detrimental to the red color stability of NO-heme, resulting in a reduced consumer acceptability of cured meat. Moreover, sausages with 0.2–0.7% CaLac presented more NO-heme contents than sausage with 0.2–0.7% CaCl_2_ (*p* < 0.05), which is also consistent with the changes in pH values, resulting in the higher a* values of CaLac treated sausages in comparison with CaCl_2_ treated sausages.

#### 3.2.3. TBARS Values and Residual Nitrite

In some cases, lipid oxidation can induce severe quality deteriorations in meat products, such as meat discoloration and undesirable flavor [31], which is detrimental to the final sensory evaluation by consumers. A marked interaction of salt type × salt concentration × storage time was observed for TBARS values. The addition of 0.7% CaCl_2_ significantly increased the TBARS values of sausages during storage for seven days (Table 4). However, sausages with all CaLac additions showed little changes (*p* > 0.05) in TBARS values with storage time. In contrast to CaLac addition, 0.7% CaCl_2_ treated sausages exhibited greater (*p* < 0.05) TBARS values than the other levels of CaCl_2_ by the end of storage. All these data suggest that high levels of CaCl_2_ addition promoted the lipid oxidation of sausages to a greater extent than CaLac during storage. There was evidence that low pH values favored the lipid oxidation of beef [32]. On the other hand, chloride ions were the components responsible for the pro-oxidant action in a model system of phosphatidylcholine liposomes [31]. Consequently, CaLac provided better oxidative stability than CaCl_2_ for sausages during storage in the present study.

A significant salt type × salt concentration × storage time interaction was also evident for residual nitrite. Sausages with both calcium salts at each level, except 0.7% CaCl_2_, resulted in a pronounced decline (*p* < 0.05) in residual nitrite contents over time (Table 4). A significant decrease in residual nitrite was also observed with elevated levels of CaCl_2_ and CaLac on either day 0 or day 7. Given that the threshold of residual nitrite in meat products is 30 ppm (National Food Safety Standard of China GB 2760-2014), a high level of CaCl_2_ (0.4–0.7%) or CaLac (0.7%) addition could accelerate nitrite reduction in sausages. At the end of storage, 0.2–0.7% CaLac treated sausages showed greater residual nitrite than 0.2–0.7% CaCl_2_ treated sausages. This corresponds with the lower pH detected in CaCl_2_ treated sausages compared to CaLac treated sausages. Honikel [25] indicated that a higher pH value retarded the reduction of nitrite concentrations during storage.

#### 3.2.4. Texture-profile Analysis (TPA)

There was a significant interaction (*p <* 0.05) between salt type and salt concentration for hardness, springiness, cohesiveness, gumminess, chewiness, and resilience. All TPA values of sausages increased significantly with elevated additions of CaCl_2_ (Table 5). Similarly, sausages with CaLac also resulted in a trend toward increases in these texture profiles, with the exception of springiness. Moreover, 0.4–0.7% CaCl_2_ treated sausages exhibited higher (*p* < 0.05) hardness, springiness, cohesiveness, gumminess, chewiness, and resilience values in relation to 0.4–0.7% CaLac treated sausages, indicating firmer texture. Generally, the moisture content is one of main factors that impact the final texture profile of meat products [33]. For instance, Damodaran [34] stated that a calcium addition to meats affected protein network restructuring, which caused water molecules to be retained, and thus decreased hardness. However, the CaCl_2_ and CaLac additions in this study reduced the moisture content of sausages as a consequence of increasing cooking loss. For this reason, an increase in hardness, springiness, adhesiveness, and chewiness values was observed. Similar CaCl_2_ or CaLac enhancements in texture profiles (hardness, chewiness, etc.) of different sausage types were also reported by Cáceres et al. [7] and Horita et al. [35].

## 4. Conclusions

The addition of various concentrations of CaCl_2_ and CaLac led to substantial changes in the physicochemical quality of beef sausages during the manufacturing and storage, which should be of interest to sausage manufacturers who may be considering alginate casing for co-extruded sausages where Ca salts are used. With increasing concentrations (0–0.7%), both calcium salts resulted in a deleterious effect on the quality of raw chopped batter and cooked sausages during manufacturing and storage, especially for the additive level of 0.7%. As the result of a better color and oxidative stability and improved texture properties, 0.2–0.4% CaLac would be better than CaCl_2_ in the production of cured beef sausages where exposure to Ca may occur, such as co-extruded alginate casings. However, it should be noted that co-extruded casings utilize Ca salts for surface application to the casings, whereas the Ca salts were added to the sausage formulation for this study to better assess the performance of Ca contact with the product. Future research is needed to investigate the influences on co-extruded sausage quality attributes by adding other potential calcium salts (e.g., calcium sorbate, calcium acetate, etc.) that might be applied to alginate casings.

## Figures and Tables

**Table 1:** Effects of calcium salt types, concentrations, and processing treatments on the pH values during the processing of beef sausages.

Calcium Salts	Processing	Salt Concentrations (%)				SE ^e^	*p*-Value
		0	0.2	0.4	0.7		
CaCl_2_	Ground beef	5.63 ^aly^	5.63 ^alx^	5.63 ^alx^	5.63 ^alx^	0.01	<0.001
	Chopped batter	5.55 ^alz^	5.50 ^bmz^	5.49 ^bmy^	5.41 ^cmy^		
	Cooked sausage	5.83 ^alx^	5.56 ^bmy^	5.46 ^cmz^	5.31 ^dmz^		
CaLac	Ground beef	5.63 ^aly^	5.63 ^aly^	5.63 ^aly^	5.63 ^alx^		
	Chopped batter	5.55 ^alz^	5.52 ^blz^	5.51 ^blz^	5.50 ^clz^		
	Cooked sausage	5.83 ^alx^	5.83 ^alx^	5.69 ^blx^	5.54 ^cly^		

^a–d^ Means within the same calcium salt type and processing treatment with different letters differ at *p* < 0.05. ^l,m^ Means within the same calcium salt concentration and processing treatment with different letters differ at *p* < 0.05. ^x–z^ Means within the same calcium salt concentration and calcium salt type with different letters differ at *p* < 0.05. ^e^ SE: Standard error.

**Table 2:** Effects of calcium salt types, concentrations, and processing treatments on the meat color changes during the processing of beef sausages.

Traits	Calcium Salts	Processing	Salt Concentrations (%)				SE ^d^	*p*-Value
			0	0.2	0.4	0.7		
L*	CaCl_2_	Ground beef	51.61 ^alz^	51.61 ^alz^	51.61 ^alz^	51.61 ^alz^	0.33	0.007
		Chopped batter	56.48 ^aly^	56.18 ^amy^	56.04 ^aly^	56.08 ^aly^		
		Cooked sausage	62.33 ^bclx^	63.30 ^alx^	62.92 ^ablx^	61.95 ^clx^		
	CaLac	Ground beef	51.61 ^alz^	51.61 ^alz^	51.61 ^alz^	51.61 ^alz^		
		Chopped batter	56.48 ^bly^	57.30 ^aly^	55.47 ^cly^	55.79 ^bcly^		
		Cooked sausage	62.33 ^ablx^	61.82 ^bmx^	62.81 ^alx^	62.21 ^ablx^		
a*	CaCl_2_	Ground beef	24.43 ^alx^	24.43 ^alx^	24.43 ^alx^	24.43 ^alx^	0.19	<0.001
		Chopped batter	9.07 ^alz^	8.34 ^cmz^	8.42 ^bclz^	8.77 ^ablz^		
		Cooked sausage	13.55 ^aly^	13.40 ^aly^	12.57 ^bmy^	11.48 ^cmy^		
	CaLac	Ground beef	24.43 ^alx^	24.43 ^alx^	24.43 ^alx^	24.43 ^alx^		
		Chopped batter	9.07 ^alz^	8.85 ^alz^	7.96 ^bmz^	7.66 ^bmz^		
		Cooked sausage	13.55 ^aly^	13.65 ^aly^	13.41 ^aly^	13.40 ^aly^		
b*	CaCl_2_	Ground beef	24.70 ^alx^	24.70 ^alx^	24.70 ^alx^	24.70 ^alx^	0.22	0.020
		Chopped batter	20.21 ^aly^	19.75 ^aly^	19.96 ^aly^	19.75 ^aly^		
		Cooked sausage	12.40 ^alz^	12.45 ^alz^	12.34 ^alz^	12.37 ^alz^		
	CaLac	Ground beef	24.70 ^alx^	24.70 ^alx^	24.70 ^alx^	24.70 ^alx^		
		Chopped batter	20.21 ^bly^	20.11 ^bly^	18.85 ^amy^	18.61 ^amy^		
		Cooked sausage	12.40 ^alz^	12.53 ^alz^	12.47 ^alz^	12.32 ^alz^		
Chroma	CaCl_2_	Ground beef	34.74 ^alx^	34.74 ^alx^	34.74 ^alx^	34.74 ^alx^	0.28	<0.001
		Chopped batter	22.15 ^aly^	21.44 ^bly^	21.66 ^bly^	21.61 ^bly^		
		Cooked sausage	18.37 ^alz^	18.29 ^alz^	17.62 ^bmz^	16.88 ^cmz^		
	CaLac	Ground beef	34.74 ^alx^	34.74 ^alx^	34.74 ^alx^	34.74 ^alx^		
		Chopped batter	22.15 ^aly^	21.97 ^aly^	20.47 ^bmy^	20.12 ^bmy^		
		Cooked sausage	18.37 ^alz^	18.53 ^alz^	18.32 ^alz^	18.21 ^alz^		
Hue	CaCl_2_	Ground beef	45.31 ^aly^	45.31 ^aly^	45.31 ^aly^	45.31 ^alz^	0.23	<0.001
		Chopped batter	65.85 ^blx^	67.10 ^alx^	67.14 ^alx^	66.08 ^bmx^		
		Cooked sausage	42.46 ^clz^	42.89 ^clz^	44.50 ^blz^	47.18 ^aly^		
	CaLac	Ground beef	45.31 ^aly^	45.31 ^aly^	45.31 ^aly^	45.31 ^aly^		
		Chopped batter	65.85 ^blx^	66.25 ^bmx^	67.11 ^alx^	67.63 ^alx^		
		Cooked sausage	42.46 ^alz^	42.55 ^alz^	42.92 ^amz^	42.60 ^amz^		

^a–c^ Means within the same calcium salt type and processing treatment with different letters differ at *p* < 0.05. ^l,m^ Means within the same calcium salt concentration and processing treatment with different letters differ at *p* < 0.05. ^x–z^ Means within the same calcium salt concentration and calcium salt type with different letters differ at *p* < 0.05. ^d^ SE: Standard error.

**Table 3:** Effects of calcium salt types, concentrations, and storage time on the color of beef sausages during chilled storage.

Traits	Calcium Salts	Storage Time	Salt Concentrations (%)				SE ^e^	*p*-Value
			0	0.2	0.4	0.7		
L*	CaCl_2_	Day0	62.33 ^clz^	63.30 ^alz^	62.92 ^blz^	61.95 ^dlz^	0.12	<0.001
		Day7	64.05 ^bly^	64.20 ^bly^	64.65 ^aly^	64.11 ^bly^		
	CaLac	Day0	62.33 ^blz^	61.82 ^cmz^	62.81 ^alz^	62.21 ^blz^		
		Day7	64.05 ^bly^	63.79 ^bmy^	64.63 ^aly^	63.78 ^bmy^		
a*	CaCl_2_	Day0	13.55 ^aly^	13.40 ^amy^	12.57 ^bmz^	11.48 ^cmz^	0.09	0.047
		Day7	13.76 ^aly^	13.63 ^amy^	13.11 ^bmy^	12.33 ^cmy^		
	CaLac	Day0	13.55 ^alz^	13.65 ^alz^	13.41 ^alz^	13.40 ^alz^		
		Day7	13.76 ^ably^	13.92 ^aly^	13.87 ^ably^	13.65 ^bly^		
b*	CaCl_2_	Day0	12.40 ^alz^	12.45 ^aly^	12.35 ^aly^	12.37 ^aly^	0.05	0.012
		Day7	12.62 ^aly^	12.49 ^abmy^	12.41 ^bmy^	12.26 ^cmy^		
	CaLac	Day0	12.40 ^ablz^	12.53 ^alz^	12.47 ^alz^	12.32 ^blz^		
		Day7	12.62 ^bly^	12.77 ^aly^	12.78 ^aly^	12.68 ^ably^		
Chroma	CaCl_2_	Day0	18.37	18.29	17.62	16.88	0.06	
		Day7	18.67	18.49	18.05	17.39		
		Means	18.52 ^al^	18.39 ^am^	17.84 ^bm^	17.14 ^cm^		<0.001
	CaLac	Day0	18.37	18.53	18.32	18.21		
		Day7	18.67	18.89	18.71	18.80		
		Means	18.52 ^bl^	18.71 ^al^	18.52 ^bl^	18.51 ^bl^	0.06	<0.001
Hue	CaCl_2_	Day0	42.46 ^cly^	42.89 ^cly^	44.50 ^bly^	47.18 ^aly^	0.21	0.002
		Day7	42.53 ^cly^	42.51 ^cly^	43.43 ^blz^	44.83 ^alz^		
	CaLac	Day0	42.46 ^aly^	42.55 ^aly^	42.92 ^amy^	42.60 ^amy^		
		Day7	42.53 ^bly^	42.53 ^bly^	43.12 ^aly^	42.44 ^bmy^		

^a–d^ Means within the same calcium salt type and storage time with different letters differ at *p* < 0.05. ^l,m^ Means within the same calcium salt concentration and storage time with different letters differ at *p* < 0.05. ^y,z^ Means within the same calcium salt concentration and calcium salt type with different letters differ at *p* < 0.05. ^e^ SE: Standard error.

**Table 4:** Effects of calcium salt types, concentrations, and storage time on the TBARS values and residual nitrite content of beef sausages during chilled storage.

Traits	Calcium Salts	Storage Time	Salt Concentrations (%)				SE ^c^	*p*-Value
			0	0.2	0.4	0.7		
TBARS (mg MDA/kg)	CaCl_2_	Day0	0.35 ^aly^	0.33 ^aly^	0.30 ^amy^	0.32 ^alz^	0.02	0.037
		Day7	0.31 ^bly^	0.30 ^bly^	0.34 ^bly^	0.42 ^aly^		
	CaLac	Day0	0.35 ^aly^	0.35 ^aly^	0.35 ^aly^	0.37 ^aly^		
		Day7	0.31 ^aly^	0.32 ^aly^	0.33 ^aly^	0.32 ^amz^		
Residual nitrite (mg/kg)	CaCl_2_	Day0	60.61 ^aly^	40.49 ^bmy^	24.84 ^cmy^	13.77 ^dmy^	1.10	0.014
		Day7	52.60 ^alz^	32.65 ^bmz^	16.73 ^cmz^	12.29 ^dmy^		
	CaLac	Day0	60.61 ^aly^	52.81 ^bly^	42.94 ^cly^	28.86 ^dly^		
		Day7	52.60 ^alz^	47.82 ^blz^	36.78 ^clz^	22.10 ^dlz^		

^a–d^ Means within the same calcium salt type and storage time with different letters differ at *p* < 0.05. ^l,m^ Means within the same calcium salt concentration and storage time with different letters differ at *p* < 0.05. ^y,z^ Means within the same calcium salt concentration and calcium salt type with different letters differ at *p* < 0.05. ^c^ SE: Standard error.

**Table 5:** Effects of calcium salt types and concentrations on the texture profile of beef sausages during chilled storage.

Traits	Calcium Salts	Salt Concentrations (%)				SE ^e^	*p*-Value
		0	0.2	0.4	0.7		
Hardness (N)	CaCl_2_	70.48 ^dy^	83.05 ^cy^	94.03 ^by^	111.79 ^ay^	1.79	<0.001
	CaLac	70.48 ^cy^	80.63 ^by^	79.89 ^bz^	98.58 ^az^		
Springiness (mm)	CaCl_2_	0.86 ^cy^	0.87 ^by^	0.88 ^by^	0.89 ^ay^	<0.01	0.001
	CaLac	0.86 ^ay^	0.87 ^ay^	0.86 ^az^	0.87 ^az^		
Cohesiveness	CaCl_2_	0.54 ^dy^	0.57 ^cy^	0.63 ^by^	0.69 ^ay^	<0.01	<0.001
	CaLac	0.55 ^cy^	0.57 ^aby^	0.56 ^bcz^	0.59 ^az^		
Gumminess (N)	CaCl_2_	39.47 ^dy^	48.05 ^cy^	58.97 ^by^	77.84 ^ay^	1.44	<0.001
	CaLac	39.47 ^cy^	47.77 ^by^	45.84 ^bz^	60.32 ^az^		
Chewiness (N)	CaCl_2_	34.56 ^dy^	41.93 ^cy^	52.07 ^by^	69.31 ^ay^	1.22	<0.001
	CaLac	34.56 ^dy^	41.95 ^by^	38.80 ^cz^	53.37 ^az^		
Resilience	CaCl_2_	0.25 ^dy^	0.26 ^cy^	0.30 ^by^	0.35 ^ay^	<0.01	<0.001
	CaLac	0.25 ^cy^	0.26 ^aby^	0.26 ^bz^	0.27 ^az^		

^a–d^ Means within the same calcium salt type with different superscript letters differ at *p* < 0.05. ^y,z^ Means within the same calcium salt concentration with different superscript letters differ at *p* < 0.05. ^e^ SE: Standard error.

## Data Availability

The data generated from the study is clearly presented and discussed in the manuscript.
